# Addison's Disease: A Diagnosis Easy to Overlook

**DOI:** 10.7759/cureus.13364

**Published:** 2021-02-15

**Authors:** Ana Margarida Mosca, Mariana Barbosa, Rosário Araújo, Maria Joana Santos

**Affiliations:** 1 Internal Medicine, Hospital de Braga, Braga, PRT; 2 Endocrinology, Hospital de Braga, Braga, PRT

**Keywords:** addison disease, hyponatremia, hyperpigmentation

## Abstract

Addison's disease is a rare and potentially life-threatening clinical condition that often presents with an insidious onset of nonspecific symptoms and signs, frequently resulting in a significant delay in diagnosis. Clinical presentation usually includes fatigue and electrolyte imbalance disorders such as hyponatremia. However, specific diagnostic features, such as hyperpigmentation, should raise clinical suspicion.

This case report describes a 43-year-old Caucasian male who presented with general malaise, fatigue, anorexia, and weight loss (7 Kg in four weeks). On physical examination, he was found to have severe hyperpigmentation of the skin and mucosal surfaces as well as hypotension. Laboratory tests revealed hypoosmolar hyponatremia and serum potassium levels in the upper limit of normal. Findings of high serum adrenocorticotropic hormone (ACTH) and renin, as well as low cortisol and aldosterone levels, helped establish a diagnosis of Addison's disease. After the initiation of treatment, the patient experienced full recovery of symptoms, normalization of hyponatremia, and improvement of hyperpigmentation.

Patients with Addison's disease have the potential to resume normal daily activities with a highly functional status. However, this condition requires lifelong follow-up and surveillance.

## Introduction

Addison's disease is a rare, potentially life-threatening clinical condition [1] that is characterized by the inability of the adrenal cortex to produce sufficient amounts of glucocorticoids and mineralocorticoids. Barthel et al. have estimated the current prevalence of the condition in Western societies to be about 100-140 cases per million [2]. This disease often manifests between the second and fourth decades of life and occurs more frequently in women than men [3].

In Western Europe, autoimmunity accounts for approximately 85% of Addison's disease diagnoses; other causes include tuberculosis and other infectious diseases, adrenal hemorrhage, and genetic disorders [1,2]. The common autoimmune form is characterized by 21-hydroxylase autoantibodies [1,4,5]; it can present as an isolated condition or be associated with other autoimmune diseases as part of an autoimmune polyglandular syndrome [5].

Patients can present with insidious onset of nonspecific symptoms, depending on the magnitude of cortisol, mineralocorticoids, and adrenal androgens deficit [6,7]. Several vague symptoms such as fatigue, anorexia, orthostatic hypotension, nausea, dizziness, and weight loss, or specific signs like salt-craving and hyperpigmentation may occur as presenting diagnostic features. Hyperpigmentation is usually generalized but is more evident in palmar creases, buccal mucosa, vermilion border of the lips, and around recent scars and nipples [3,7]. Patients are often hyponatremic, hyperkalemic, and acidotic [6].

The diagnosis is established through a biochemical assessment of the glucocorticoid hypothalamus-pituitary-adrenal (HPA) axis, as well as mineralocorticoid function. Prompt diagnosis is critical so that early and adequate treatment can be initiated and patients can be followed up appropriately [8].

## Case presentation

A 43-year-old caucasian male with a past medical history of hypertension was admitted to the emergency department with symptoms of general malaise, fatigue, anorexia, and weight loss of 7 Kg in the past four weeks. The patient had no nausea, vomiting, abdominal pain, or neurological complaints. A month earlier, he had been admitted to the emergency department and diagnosed with iatrogenic hypotonic hyponatremia caused by an antihypertensive drug (a thiazide diuretic), which had been stopped after this episode. He was not under any other drugs. Hyperpigmentation was not described and his last blood chemistry analysis at that time had revealed hyponatremia: sodium of 125 mmol/L (normal range: 135-145 mmol/L).

**Figure 1:**
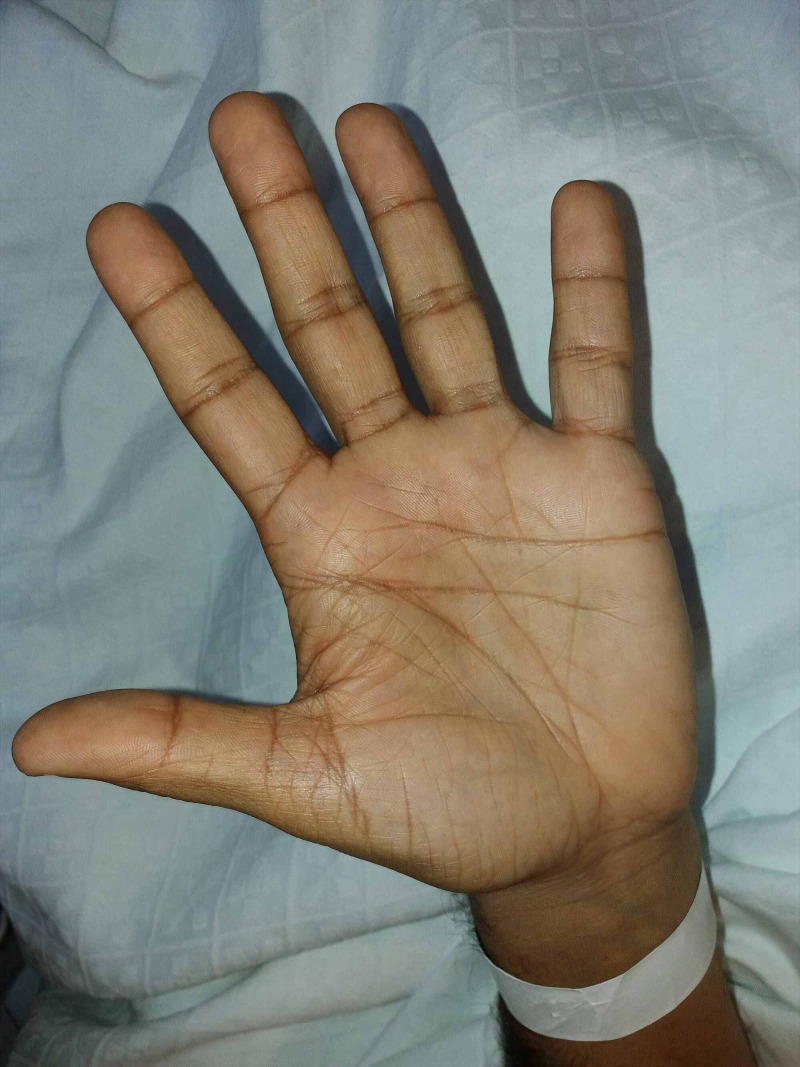
Addisonian skin hyperpigmentation involving palmar creases

**Figure 2:**
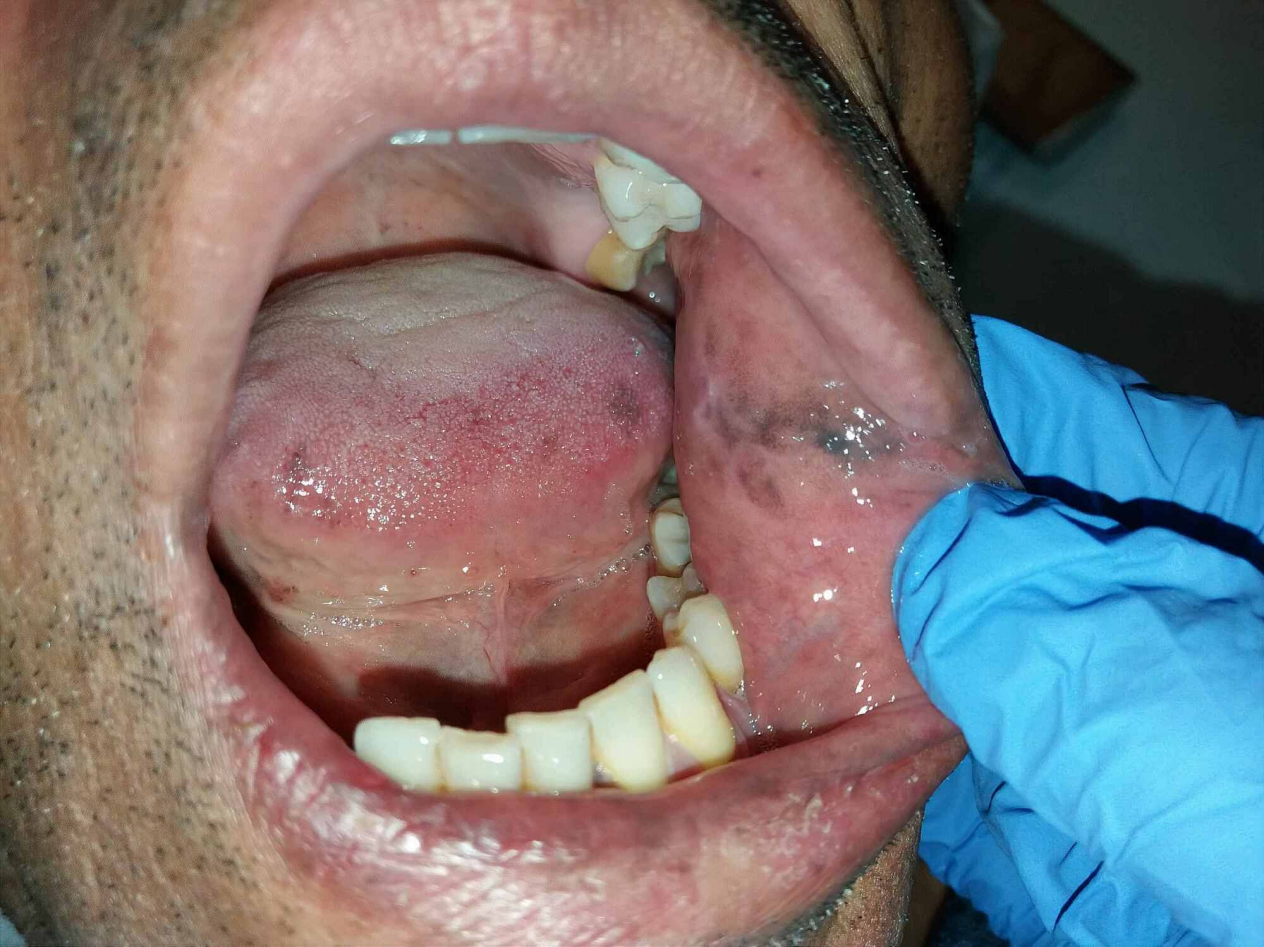
Addisonian pigmentation of buccal mucosa

On physical examination at the current presentation, the patient had severe hyperpigmentation of skin and mucosal surfaces (Figure 1, Figure 2) and hypotension. He had no signs of acute abdomen, fever, hypoglycemia, or severe dehydration.

Laboratory tests showed persistent hypotonic hyponatremia (sodium: 125 mmol/L, serum osmolality: <275 mOsm/kg) and serum potassium levels in the upper range of normal: 5.5 mmol/L (normal range: 3.5-5.5 mmol/L). Sodium urinary excretion was elevated (≥40 mmol/L) and renal function was normal (creatinine: 1.0 mg/dL). He had no elevation of the inflammatory marker C-reactive protein (<2.9 mg/L) and thyrotropin (TSH) level was 1.22 uUI/mL (normal range: 0.35-3.74 uUI/mL), which ruled out hypothyroidism as the cause for hyponatremia.

Given the presence of hyperpigmentation and hypotension, primary adrenal insufficiency was considered as a possible diagnostic hypothesis. Further investigation showed elevated basal adrenocorticotropic hormone (ACTH) levels: 1,140 pg/mL (normal range: 0-11 pg/mL) and low cortisol: 4.1 µg/dL. Serum renin concentration was 233.1 µUI/mL (normal range: 4.4-46.1 µUI/mL) and serum aldosterone was low (2.3 ng/dL). These findings helped to confirm the clinical suspicion of Addison's disease. Abdomino-pelvic CT scan was performed, which ruled out signs of infectious, hemorrhagic, infiltrating, or metastatic involvement of the adrenals. Also, the patient was HIV-negative. Given the likelihood of an autoimmune etiology, other potentially associated endocrine autoimmune disorders were excluded, namely diabetes (fasting glucose was 85 mg/dL), thyroid disease (TSH was normal as mentioned previously), or calcium metabolism alterations [corrected calcium was 9.0 mg/dL (normal range: 8.5-10 mg/dL)].

The patient was started on intravenous infusion of sodium chloride 0.9% solution (infusion rate: 63 mL/h). Glucocorticoid replacement therapy was initiated with intravenous hydrocortisone (100 mg bolus at first, followed by 8/8h administrations) with tapering of the steroid dose in the following two days, until an oral maintenance dose of hydrocortisone (15 mg + 10 mg + 5 mg) was reached. Mineralocorticoid replacement with oral fludrocortisone 0.1 mg daily was started when the saline infusion was stopped. The treatment for adrenal insufficiency led to complete elimination of symptoms, normalization of hyponatremia, and improvement of hyperpigmentation. At the latest follow-up (15 months since diagnosis), the patient was found asymptomatic under maintenance therapy with hydrocortisone 25 mg/day (12.6 mg/m^2^) divided into three-times-a-day dosing (the highest on awakening), plus fludrocortisone 0.1 mg/day.

## Discussion

In this article, we presented a case of a challenging diagnosis of Addison's disease. The delay experienced in diagnosis is frequently described in the literature and is a direct consequence of nonspecific clinical signs and symptoms in the early stages of the disease [1]. The final diagnosis is often made when patients present with symptoms of acute adrenal insufficiency, the so-called adrenal crisis [1], which was not the case with our patient. Nevertheless, he presented with chronic features for several weeks with gradual clinical deterioration, and we speculate that the previous episode of hyponatremia was already associated with primary adrenal insufficiency and was possibly aggravated by the anti-hypertensive medication.

Hypotonic hyponatremia can occur in the setting of primary polydipsia, advanced renal impairment, thiazide diuretic use, reduced effective arterial blood volume, syndrome of inappropriate antidiuretic hormone (ADH) secretion, hypothyroidism, and adrenal insufficiency, among other etiologies. In fact, although Addison's disease is a rare cause of hyponatremia, it should be considered in the diagnostic workup related to electrolyte abnormality, especially if other specific features are present [8].

In our case, apart from hypoosmolar hyponatremia, the patient presented with skin and mucosal hyperpigmentation. In Addison's disease, hyponatremia results from aldosterone deficiency (leading to renal sodium loss) and water retention (via the increased release of ADH) in response to a reduction in systemic blood pressure and cardiac output [9]. The cause of hyperpigmentation in this setting has long been debated but is thought to reflect increased stimulation of melanocortin 1 receptor by ACTH itself and possibly by melanocyte-stimulating-hormone, which also originates from the pro-hormone peptide pro-opiomelanocortin (POMC), the ACTH precursor that is strongly elevated in Addison's disease due to a lack of cortisol-mediated feedback inhibition of the HPA axis [3,8,9]. On the other hand, our patient denied salt-craving and did not present with definite hyperkalemia, although potassium levels were in the upper level of normal. Indeed, even though these two clinical features are described in the literature, they are not as common as hyperpigmentation and hyponatremia [8].

As mentioned above, autoimmune adrenalitis accounts for the majority of Addison's disease cases, but other etiologies should also be taken into account. In the present case, clinical history and laboratory/imaging findings helped us exclude less frequent causes, such as infectious diseases like HIV, as well as infiltrative, metastatic, or hemorrhagic adrenal involvement [8].

The treatment for Addison's disease consists of lifelong steroid replacement (both glucocorticoids and mineralocorticoids) [1,2,7]. Glucocorticoid replacement includes oral prednisone or hydrocortisone titrated to the lowest tolerated dose that controls symptoms and minimizes adverse effects. Mineralocorticoids are replaced with fludrocortisone to keep the plasma renin level in the upper limit of the normal range and to maintain water and electrolyte balance [2,7].

Although this entity is associated with significant morbidity rates, once the diagnosis is made, it can be easily treated and patients can return to a highly functional status [1,2]. Raising awareness among patients is also of vital importance to enable self-adjustment regarding replacement therapy and crisis prevention during concurrent illnesses or injury [1].

## Conclusions

It is important that health professionals gain awareness about the clinical features of Addison's disease so that prompt diagnosis can be made and early treatment initiated. We also lay stress on the value of a complete clinical history and a thorough physical examination during the evaluation of patients presenting with hyponatremia.
